# Nutritional Energy Stimulates NAD^+^ Production to Promote Tankyrase-Mediated PARsylation in Insulinoma Cells

**DOI:** 10.1371/journal.pone.0122948

**Published:** 2015-04-13

**Authors:** Linlin Zhong, Tsung-Yin J. Yeh, Jun Hao, Nasim Pourtabatabaei, Sushil K. Mahata, Jianhua Shao, Steven D. Chessler, Nai-Wen Chi

**Affiliations:** 1 Research Service, VA San Diego Healthcare System, San Diego, CA 92161, United States of America; 2 Department of Medicine, University of California San Diego, La Jolla, CA 92093, United States of America; 3 Department of Pathology, Hebei Medical University, Shijiazhuang, China; 4 Department of Pediatrics, University of California San Diego, La Jolla, CA 92093, United States of America; 5 Department of Medicine, University of California Irvine, Irvine, CA 92697, United States of America; Johns Hopkins University, UNITED STATES

## Abstract

The poly-ADP-ribosylation (PARsylation) activity of tankyrase (TNKS) regulates diverse physiological processes including energy metabolism and *wnt*/β-catenin signaling. This TNKS activity uses NAD^+^ as a co-substrate to post-translationally modify various acceptor proteins including TNKS itself. PARsylation by TNKS often tags the acceptors for ubiquitination and proteasomal degradation. Whether this TNKS activity is regulated by physiological changes in NAD^+^ levels or, more broadly, in cellular energy charge has not been investigated. Because the NAD^+^ biosynthetic enzyme nicotinamide phosphoribosyltransferase (NAMPT) *in vitro* is robustly potentiated by ATP, we hypothesized that nutritional energy might stimulate cellular NAMPT to produce NAD^+^ and thereby augment TNKS catalysis. Using insulin-secreting cells as a model, we showed that glucose indeed stimulates the autoPARsylation of TNKS and consequently its turnover by the ubiquitin-proteasomal system. This glucose effect on TNKS is mediated primarily by NAD^+^ since it is mirrored by the NAD^+^ precursor nicotinamide mononucleotide (NMN), and is blunted by the NAMPT inhibitor FK866. The TNKS-destabilizing effect of glucose is shared by other metabolic fuels including pyruvate and amino acids. NAD^+^ flux analysis showed that glucose and nutrients, by increasing ATP, stimulate NAMPT-mediated NAD^+^ production to expand NAD^+^ stores. Collectively our data uncover a metabolic pathway whereby nutritional energy augments NAD^+^ production to drive the PARsylating activity of TNKS, leading to autoPARsylation-dependent degradation of the TNKS protein. The modulation of TNKS catalytic activity and protein abundance by cellular energy charge could potentially impose a nutritional control on the many processes that TNKS regulates through PARsylation. More broadly, the stimulation of NAD^+^ production by ATP suggests that nutritional energy may enhance the functions of other NAD^+^-driven enzymes including sirtuins.

## Introduction

Nicotinamide adenine dinucleotide (NAD^+^) is the obligatory co-substrate for diverse post-translational modifications mediated by enzymes like poly-ADP-ribose polymerases (PARPs) and sirtuins (SIRTs)[1]. Mechanistically, PARPs and SIRT4 transfer the ADP-ribose moiety from NAD^+^ to substrate proteins to generate poly-ADP-ribosylation (PARsylation) and mono-ADP-ribosylation respectively, whereas SIRT1 and several other sirtuins transfer the ADP-ribose moiety to the acetyl group of substrate proteins to effect deacetylation. All these NAD^+^-consuming reactions release nicotinamide as a byproduct. By modulating gene transcription as well as protein function and turnover, NAD^+^-dependent enzymes regulate diverse physiological processes ranging from aging to energy homeostasis [1].

Tankyrase (TNKS) is a PARP that is expressed in many cell types and in multiple subcellular locales [2]. Its modular structure includes five clusters of ankyrin (ANK) repeats in the N-terminal ANK domain and a C-terminal PARP catalytic domain. The ANK domain serves as a multivalent scaffold that recruits substrates for the PARP domain. TNKS-mediated PARsylation modulates diverse physiological processes. TNKS for instance can bind and PARsylate TRF1, a telomere-binding protein that normally acts to shorten telomeres. PARsylation by TNKS dissociates TRF1 from telomeres, allowing the telomeres to expand [3]. TNKS can also bind and PARsylate Axin, which normally inhibits *wnt*/β-catenin signaling and also promotes glucose uptake through the transporter GLUT4 [4,5]. Because PARsylation by TNKS leads to axin degradation, the TNKS inhibitor XAV939 can stabilize axin to potentiate its effect on *wnt* signaling and glucose uptake [4,5]. Lastly, insulin-responsive amino peptidase (IRAP) is also a TNKS substrate *in vitro*. This transmembrane protein serves as an adaptor that connects TNKS with GLUT4-containing vesicles to regulate the trafficking of the latter [6]. Quantitatively, the most important TNKS substrate is the enzyme itself [7]. AutoPARsylation earmarks TNKS for ubiquitination and subsequent proteasomal degradation [8]. This poly-ADP-ribose (PAR)-directed process is initiated by the E3 ubiquitin ligase RNF146, which uses a WWE domain to bind PAR [9].

Little is known about how the PARsylating activity of TNKS is regulated. In cultured adipocytes, insulin signaling has been reported to either augment [10] or attenuate TNKS catalysis [5] through the *ras*/MAPK pathway or the PI3K-Akt pathway, respectively. The possibility of TNKS regulation by NAD^+^ bioavailability has not been investigated. However, this notion of TNKS catalysis being rate-limited by NAD^+^ availability seems plausible because physiological NAD^+^ concentrations (0.2 to 0.7 mM [11,12]) are below the *k*_m_ of TNKS (0.9–1.5 mM NAD^+^ [3,13]). Indeed, in pathological states where NAD^+^ is depleted by genotoxin exposure, TNKS autoPARsylation is attenuated [7].

Mammalian cells can synthesize NAD^+^ from tryptophan, but this *de novo* pathway is quantitatively less important than the alternative pathway that salvages nicotinamide to regenerate NAD^+^ [1,14]. The rate-limiting step in the salvage pathway is the conversion of nicotinamide to nicotinamide mononucleotide (NMN) by nicotinamide phosphoribosyltransferase (NAMPT). NMN is then converted by NMN adenylyltransferase (NMNAT) to NAD^+^. NAMPT gene transcription is regulated by circadian and nutritional cues, leading to corresponding oscillations of cellular NAD^+^ content [12]. Both caloric restriction and glucose restriction can increase NAMPT gene expression in muscle to raise NAD^+^ content [1,15]. Modulation of NAMPT activity at the catalytic level has not been demonstrated in a cellular context. However, biochemical studies have consistently shown activation of NAMPT by ATP *in vitro* [16–19], suggesting that cellular energy charge could potentially boost NAMPT-mediated NAD^+^ production.

The energy charge of insulin-secreting ß cells in pancreatic islets is highly dependent on ambient glucose levels. Owing to their expression of the high-*k*_m_ (low-affinity) hexokinase isoform glucokinase, ß cells can respond to increases in ambient glucose levels by raising glycolytic flux to augment mitochondrial ATP production [20]. Their expression of NAMPT [21] suggests that NAD^+^ production in ß cells may be responsive to ATP levels and therefore exhibits augmentation by the energetic effect of glucose. Indeed, circumstantial evidence suggests that ß cells harbor more NAD^+^ in the fed state than upon fasting or caloric restriction (CR). First, the NAD^+^-driven ADP-ribosylation of glutamate dehydrogenase by SIRT4 in ß cells is greater in *ad-lib* fed mice than in CR mice despite comparable SIRT4 protein levels [22]. Secondly, SIRT1-mediated inhibition of UCP2 expression in the ß cells is also more active in fed mice than fasted mice despite comparable SIRT1 protein levels [23]. Lastly and albeit not measured specifically in the ß cells, pancreatic NAD^+^ content is higher in fed mice than fasted mice [23]. These observations collectively support the hypothesis that ß cells, in response to the energetic effect of nutrients and particularly glucose, may increase NAMPT-mediated production of NAD^+^. This hypothesis predicts that nutrients, by increasing NAD^+^ bioavailability, should stimulate TNKS catalysis in ß cells. We therefore used the ß cell lines INS-1 and MIN6 to investigate the proposed nutrients → ATP → NAMPT → NAD^+^ → TNKS pathway.

## Materials and Methods

### Cell cultures and treatments

INS-1 cells were cultured in RPMI medium containing 11 mM glucose as described [24]. MIN6 [25], 3T3-L1 preadipocytes [10], and a subclone of HEK293 cells called BOSC [10] were maintained in DMEM medium containing 28 mM glucose. Several days after reaching confluence, regular media were replaced with glucose-free RPMI (Gibco Inc.) or DMEM (Mediatech Inc.) supplemented with the indicated amount of glucose and other compounds. FK866, PJ-34, and alanyl-glutamine (GlutaMAX) were from AdipoGen Inc., Inotek Corp. and Gibco Inc., respectively. XAV939 (Sigma Inc.), MG-132, and the glucokinase activator RO-28-1675 (Axon Medchem, Inc. [26]) were dissolved in DMSO at 15, 10, and 50 mM respectively for stock solutions. Fatty acids (a cocktail of linoleic acid, oleic acid, and albumin at the molar ratio of 2:1:1) and all other compounds were from Sigma Inc.

### Affinity precipitation, immunoblotting, and protein staining

For immunoblotting of whole-cell extracts, cells were lysed directly in SDS sample buffer. Alternatively, cells were lysed in the cold room in buffer A [10] supplemented with either N-ethylmaleimide (10 mM; Sigma Inc.) to inhibit deubiquitination (for GST-S5a pulldown) or ADP-ribose (1 mM; Sigma Inc.) and ADP-HPD (1 μM; Calbiochem Inc.) to inhibit PAR hydrolysis (for GST-IRAP and GST-WWE pulldown). After removing insoluble proteins by microcentrifugation (25,000 *g* for 30 min), soluble lysates were incubated with resins containing one of the following GST fusions (15–20 μg per reaction). To affinity-precipitate TNKS for anti-PAR immunoblotting, GST-IRAP_aa78-109_ [10] containing a hexapeptide sequence (RQSPDG) that binds to the ANK domain of TNKS was used. To precipitate PARsylated species, lysates were supplemented with 0.1% SDS prior to incubation with GST-WWE resins (Tulip BioLab Inc.), which contained the PAR-avid WWE domain of the E3 ubiquitin ligase RNF146 as described in [9]. As negative control, a mutant GST-WWE (Tulip BioLab Inc.) containing an R163A substitution that abolishes PAR binding [9] was used. To precipitate ubiquitinated species, lysates were incubated with GST fusions of the S5a subunit of the proteasome as described [27] except that sequences outside the ubiquitin-binding motifs (aa 193–307) of S5a were deleted by subcloning. After 6–14 hr incubation with the above affinity resins, the precipitates were washed 3 times in buffer A [10], denatured in SDS sample buffer for 10 min at 65°C (for anti-PAR immunoblots) or 95°C (all other figures), and resolved in either 6.5% gels or 6–10% step-gradient gels. Prior to transferring to nitrocellulose membranes (for anti-PAR immunoblotting) or PVDF membranes (all other figures), the lower portion of the gels was excised to stain GST fusions using Coomassie dye from either Sigma Inc. or G-Biosciences. Antibodies against NAMPT (clone 14A5, a gift from Dr. Oberdan Leo), RNF146 and GAPDH (both from Genetex Inc.) were used at 0.2, 1, and 0.2 μg/ml, respectively. Rabbit anti-PAR antibodies (LP96-10, a gift from Dr. Guy Poirier [28]) and anti-ubiquitin antibodies (Sigma Inc.) were used at 1:5000 dilution. Antibodies against TNKS (polyclonal H-350) and PARP1 (monoclonal C2-10) were described in [7,29]. Immunoblots were analyzed by densitometry as indicated. *P* values were calculated using Student’s unpaired two-tailed *t* tests.

### ATP assays

Postconfluent INS-1 cells grown in 24-well plates were lysed on ice by replacing media with cold 2.5% trichloroacetic acid (450 μl / well). After shaking the plates briefly at room temperature, lysates were neutralized with 150 μl of 1M HEPES (pH 8). Aliquots (7 μl) were mixed with CellTiter-Glo reagent (25 μl, Promega Inc.) and Opti-MEM medium (25 μl, Gibco Inc.) in white 96-well plates for ATP quantification following the manufacturer’s protocol. Luminescence was quantified in a microplate reader (Model M200, Tecan Inc.). *P* values were calculated using Student’s unpaired two-tailed *t* tests.

### NAD^+^ assays

Postconfluent INS-1 cells were lysed by replacing media with 1 M PCA (500 or 250 μl / well in 6- or 12-well plates, respectively) to extract NAD^+^ while destroying NADH. After neutralization with 2M KOH in 96-well plates, lysates were diluted 1:10 into 50 mM Bicine (pH 7.8). Aliquots (40 μl) were subjected to a chromogenic enzyme cycling assay where NAD^+^ serves as the cofactor for alcohol dehydrogenase [7]. Individual data points represent the average of 6 wells for each treatment (12 wells for untreated baseline), each well assayed in quadruplicate. *P* values were calculated using Student’s unpaired two-tailed *t* tests.

### Assessment of NAD^+^ fluxes

The change in cellular NAD^+^ content during a given treatment equals the difference between production and consumption. Therefore,

$$\;\;\left[{\text{final NAD}}^{\text{+}}\text{content}]\text{ = }[{\text{initial NAD}}^{\text{+}}\text{content}]\text{ + }[{\text{NAD}}^{\text{+}}\text{production}]\;–\;[{\text{NAD}}^{\text{+}}\text{consumption}\right]$$

The above can be rearranged as:

$$\;\;\left[{\text{NAD}}^{\text{+}}\text{production}]\text{ = }[{\text{final NAD}}^{\text{+}}\text{content}]\;–\;[{\text{initial NAD}}^{\text{+}}\text{content}]\text{ + }[{\text{NAD}}^{\text{+}}\text{consumption}\right]$$

During glucose treatment, when NAD^*+*^ production is blocked by FK866 (reducing the left side of the equation toward zero), the consumption can be calculated simply as the drop in NAD^*+*^ content (*i*.*e*., initial content in untreated samples minus the final content after FK866 treatment).

In DMSO-treated controls, the rate of ongoing NAD^*+*^ production can be determined from the same equation by plugging in [initial NAD^*+*^ content] in untreated samples, [final NAD^*+*^ content] after DMSO treatment, and the [NAD^*+*^ consumption] as determined earlier from FK866-treated samples.

## Results

### INS-1 cells produce NAD^+^ primarily through NAMPT-dependent pathway

**Fig 1:**
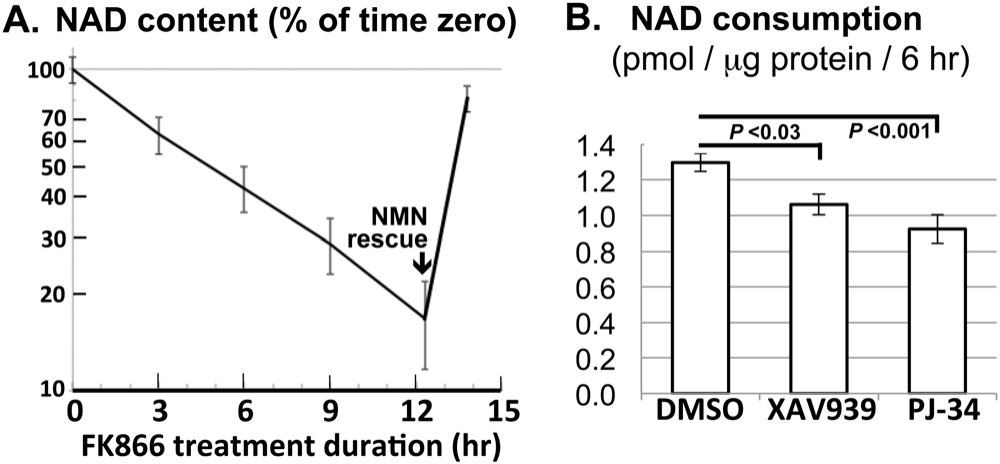
NAD^+^ analysis of INS-1 cells. **A.** Confluent cultures of INS-1 cells were treated with the NAMPT inhibitor FK866 (0.2 μM) in the presence of 11 mM glucose for the indicated duration. NMN (200 μM) was added 90 min prior to the final time point. NAD^+^ content (mean ± S.E.M.) was plotted on a semilog scale as % of pretreatment level. **B.** Confluent INS-1 cultures were treated for 6 hr with the TNKS inhibitor XAV939 (4 μM), the PARP1 inhibitor PJ-34 (12 μM), or DMSO vehicle (0.1% v/v) in the presence of 11 mM glucose. NAD^+^ consumption (mean ± S.E.M.) during the treatment was determined as described in Materials and Methods.

To assess the extent to which INS-1 cells produce NAD^+^ using the *de novo vs*. the salvage pathway, we inhibited the latter using the NAMPT inhibitor FK866 [14]. This led to progressive depletion of 85% of the initial NAD^+^ stores within 12 hours (Fig 1A). Consistent with preserved cellular viability, FK866-treated cells effectively replenished the NAD^+^ stores upon rescue by exogenous NMN (Fig 1A), the immediate NAD^+^ precursor that can enter INS-1 cells [30]. Importantly, FK866-induced NAD^+^ depletion appeared to follow first-order kinetics throughout the time course without leveling off (Fig 1A), arguing against significant *de novo* NAD^+^ production. We therefore inferred that INS-1 cells produce NAD^+^ almost exclusively through NAMPT in the salvage pathway and turn over the dinucleotide with a half-life of about 5 hours (Fig 1A). This value approximates the half-life in ovarian cancer cells [31] but exceeds the 1–2 hr half-life in 3 other cell types [32].

### NAD^+^ fluxes in INS-1 cells can be determined using FK866 treatment

Because FK866 effectively blocks NAD^+^ production, it should cause cellular NAD^+^ content to fall at a rate that equals overall NAD^+^ consumption. To confirm that FK866 treatment can be used to detect changes in NAD^+^ consumption rate, we assessed the latter in INS-1 cells treated with either XAV939 or PJ-34, inhibitors that preferentially target TNKS or PARP1 respectively [4,33]. As expected, both inhibitors significantly decreased NAD^+^ consumption rate (calculated as the difference between the content of untreated *vs*. FK866-treated cells)(Fig 1B). In subsequent experiments, we also determined NAD^+^ production rates using the strategy described in Materials and Methods. Of note, this approach modestly underestimates NAD^+^ flux since FK866-induced partial NAD^+^ depletion can potentially attenuate NAD^+^ consumption.

### NAD^+^ production in INS-1 cells is stimulated by glucose and fatty acids

**Fig 2:**
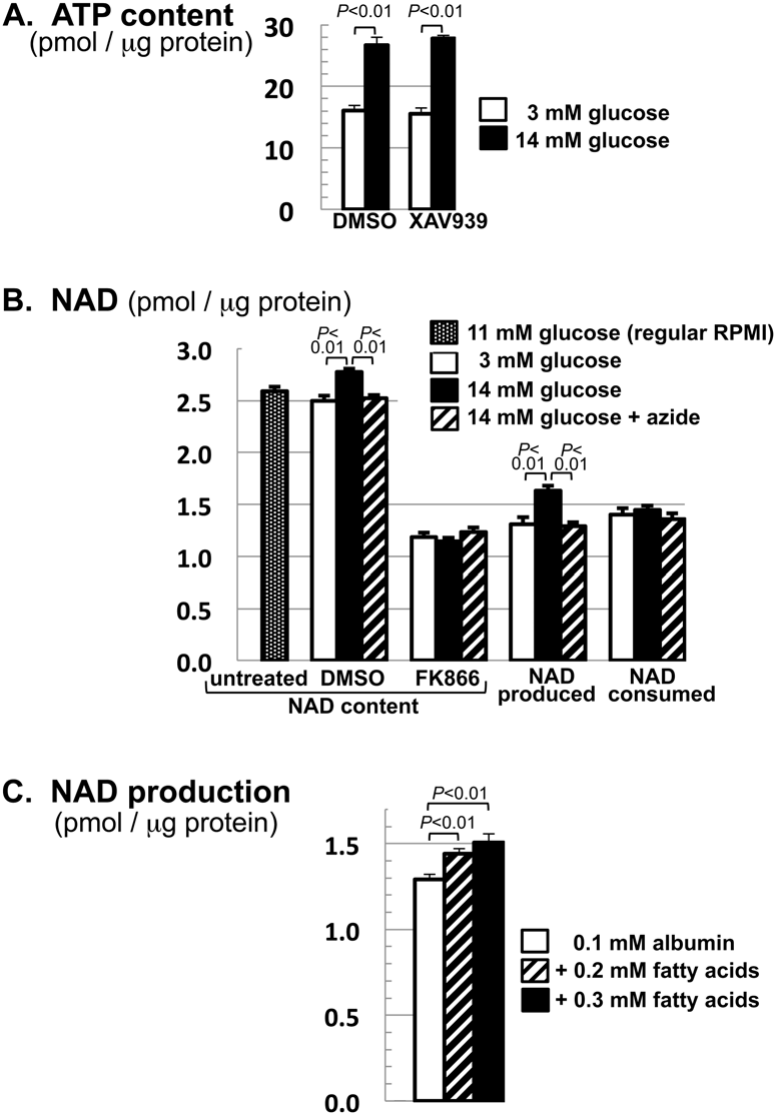
Glucose and fatty acids increase NAD^+^ production in INS-1 cells. **A.** Cells were treatment with 3 or 14 mM glucose in the presence of XAV939 (4 μM) or DMSO for 7 hr. ATP content was determined as described in Materials and Methods and normalized to protein content. **B.** Cells were grown to confluence in regular RPMI (11 mM glucose). For each 12-well plate, 2 wells served as untreated controls while the remainder were switched to fresh RPMI containing the indicated combination of glucose (3 or 14 mM), FK866 (0.2 μM), DMSO, and sodium azide (0.8 mM). Cells were lysed 7 hr later for NAD^+^ analysis. NAD^+^ contents (shown in the first 7 bars) were used to calculate the amount of NAD^+^ produced *vs*. consumed during the 7-hr treatment (shown in the last 6 bars) using the formula described in Materials and Methods. **C.** Cells were treated for 6 hr with 0, 0.2 or 0.3 mM fatty acids (a 2:1 mixture of linoleic acid and oleic acid) in the presence of 0.1 mM albumin and 3 mM glucose. NAD^+^ production during the treatment was determined as in **B.** Data shown (mean ± S.E.M.) are representative of 4 (**A**-**B**) or 2 (**C**) independent experiments.

Conventional INS-1 culture media contain 11 mM glucose. To manipulate cellular ATP levels, we exposed cells to either low (3 mM) or high (14 mM) glucose levels. These levels were used because 3 mM is below the plasma glucose level of fasted humans [34] whereas 14 mM is in the diabetic range, above the 8 mM ceiling of physiological excursion after a glucose load [35]. Moreover, these levels are in line with the convention in ß cell literature [36–38]. We found that high glucose levels not only expanded cellular ATP stores as expected (Fig 2A) but also increased NAD^+^ content (Fig 2B, 2^nd^ *vs*. 3^rd^ bar). This glucose effect on NAD^+^ content was abolished by FK866 (Fig 2B, 5^th^ *vs*. 6^th^ bar), indicating that glucose raised NAD^+^ content by augmenting NAMPT-mediated production rather than by attenuating cellular NAD^+^ consumption. This notion was born out by assessing NAD^+^ production and consumption rates separately (Fig 2B). The simplest interpretation, given the stimulation of NAMPT by ATP *in vitro* [16–19], is that glucose increased cellular energy charge to stimulate NAD^+^ production by NAMPT. To further establish energetic stimulation of NAD^+^ production, Fig 2B shows that the mitochondrial poison sodium azide prevented glucose from increasing NAD^+^ content (4^th^ bar) and its production rate (10^th^ bar), while Fig 2C shows that fatty acids as an alternative fuel recapitulated the glucose effect on NAD^+^ production. Collectively, these findings indicated that ß cells increase NAD^+^ production in response to nutritional energy.

### Glucose stimulates TNKS autoPARsylation and turnover in INS-1 cells

**Fig 3:**
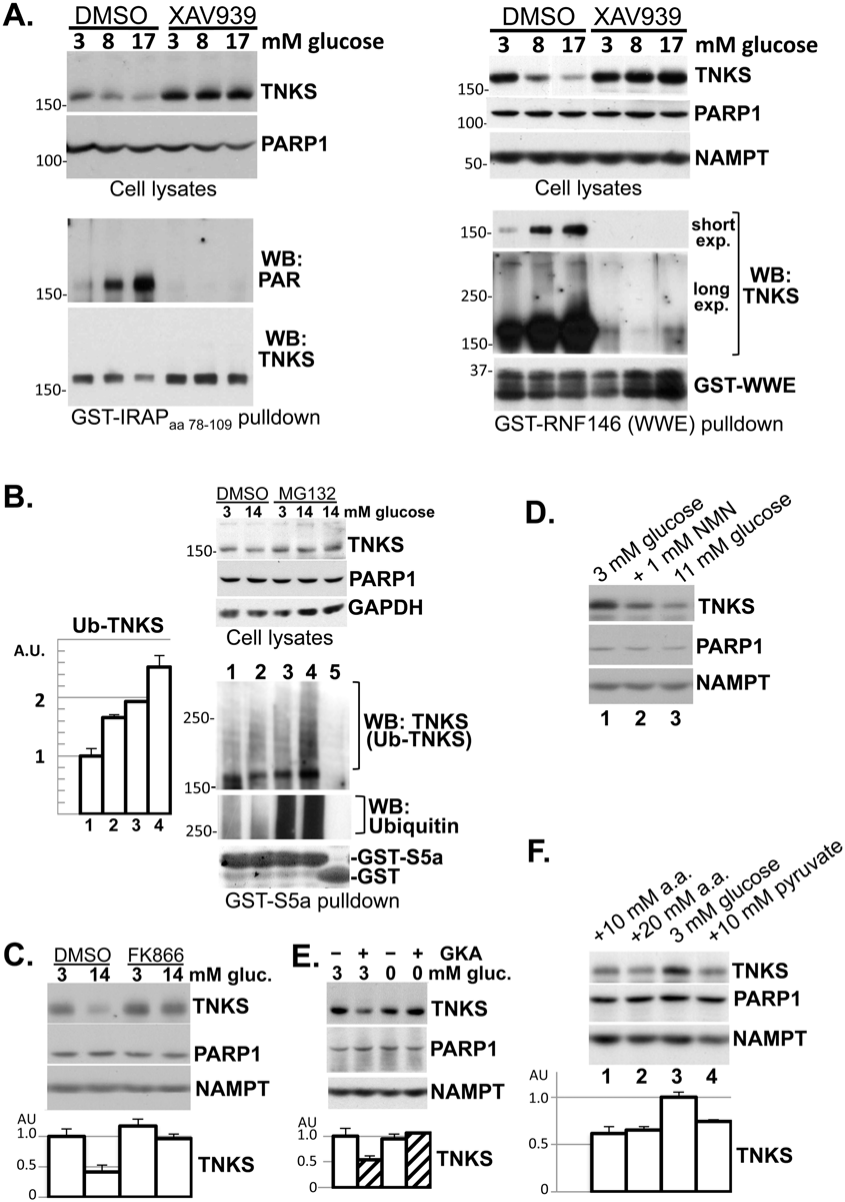
Diverse fuels promote TNKS autoPARsylation and turnover in INS-1 cells. **A. Left panels:** Cells were pretreated with XAV939 (4 μM) or DMSO in the presence of 3 mM glucose for 30 min before glucose was raised to the indicated final concentration for 5 hr. Equal aliquots of the lysates were immunoblotted (upper panels). TNKS in the remaining lysates was precipitated by incubation with resins of GST-IRAP_aa78-109_ (15 μg), which contained a hexapeptide sequence (IRAP_aa96-101_) that bound to the ANK domain of TNKS [49]. The precipitates were immunoblotted sequentially for PAR and TNKS. Each lane corresponds to a 15-cm plate. The data shown are representative of 2 independent experiments. **Right panels:** Cells were treated for 7 hr with the indicated glucose concentration in combination with either XAV939 (4 μM) or DMSO. Equal aliquots of the lysates were immunoblotted (upper panels). PARsylated species in the remaining lysates were precipitated by incubation with resins of GST fused to the WWE domain of RNF146 (20 μg) and immunoblotted for TNKS (two exposures) and RNF146 (lower panels). Each lane corresponds to a 10-cm plate. The data shown are representative of 4 independent experiments. **B.** Cells were treated with 3 or 14 mM glucose for 30 min before MG-132 (10 μM) or vehicle (DMSO) was added for another 30 min. Equal aliquots of the lysates were immunoblotted (upper panels). Ubiquitinated species in the remaining lysates were precipitated by incubation with resins of GST fused to the ubiquitin-binding motifs of S5a (20 μg, lanes 1–4), using GST as negative control (20 μg, lane 5). The precipitates were sequentially immunoblotted with anti-TNKS antibodies to detect ubiquitinated TNKS, and with anti-ubiquitin antibodies for loading control. The bar graph shows densitometer analysis of ubiquitinated TNKS (mean ± SEM; n = 3). The data shown are representative of 2 independent experiments. **C.** Cells were treated with FK866 (0.2 μM) or DMSO in the presence of 3 or 14 mM glucose. **D**. Cells were treated with 3 mM glucose (lane 1), 1 mM NMN in combination with 3 mM glucose (lane 2), or 11 mM glucose (lane 3). **E.** Cells were treated with the indicated concentration of glucose along with a glucokinase activator (GKA, 10 μM RO-28-1675 [50]) or DMSO. **F.** Cells were treated with 3 mM glucose, either alone (lane 3) or in combination with alanyl-glutamine (10 mM in lane 1; 20 mM in lane 2) or pyruvate (10 mM, lane 4). For **C-F,** the treatment duration was 6–8 hr. Each lane represents 10% of whole-cell extracts from a well in 24-well plates. The immunoblots are representative of 2–3 experiments, each performed in 2–4 replicates. The bar graphs show densitometer quantification of TNKS abundance (mean ± SEM).

To determine if glucose-stimulated NAD^+^ production is reflected in TNKS autoPARsylation, we treated INS-1 cells with various glucose levels for 5 hr and then precipitated TNKS for anti-PAR immunoblotting. Fig 3A (panels on the left) shows that glucose increased the PAR content of TNKS despite decreasing total TNKS abundance. Regardless of glucose levels, XAV939 pretreatment eliminated TNKS PARsylation (Fig 3A), confirming the specificity of the anti-PAR immunoblotting. As a complementary strategy for detecting PARsylated TNKS, we used the PAR-avid WWE domain of RNF146 [9] to isolate PARsylated species from cell lysates for anti-TNKS immunoblotting. This strategy showed that TNKS binding to the WWE domain was increased by 1-hr (S1 Fig) or 7-hr glucose treatment and was abolished by XAV939 (Fig 3A, panels on the right). Both strategies in Fig 3A detected PARsylated TNKS primarily as a band instead of a smear, consistent with prior reports involving overexpressed TNKS in other cell lines [7,39]. A limited fraction of PARsylated TNKS migrated as a trailing smear (Fig 3A, longer exposure). The elimination of this smear by XAV939 confirmed its requirement for the catalytic activity of TNKS.

To confirm that glucose-induced TNKS PARsylation led to TNKS turnover through the ubiquitin-proteasomal system, Fig 3B shows that TNKS ubiquitination was increased by 1 hr of glucose treatment, leading to a robust decrease in TNKS levels after 5–7 hr treatment (Fig 3A). The TNKS-destabilizing effect of glucose was blocked by XAV939 (Fig 3A) and FK866 (Fig 3C), indicating the requirement for TNKS autoPARsylation and NAMPT-mediated NAD^+^ production, respectively. To further implicate NAD^+^ in promoting TNKS turnover, Fig 3D shows decreased TNKS abundance in response to NMN, the immediate NAD^+^ precursor. Collectively, these data indicated that glucose promoted TNKS PARsylation and turnover by increasing NAMPT-mediated NAD^+^ production in INS-1 cells.

Glucose metabolism in INS-1 cells is rate-limited at the first step of glycolysis by glucokinase (GK), the hexokinase with the lowest glucose affinity [40]. Consequently, small molecules like RO-28-1675 that increase the glucose affinity of GK can serve as GK activators (GKAs) that increase glycolytic flux at low glucose levels [26]. Fig 3E shows that GKA decreased TNKS abundance in the presence of 3 mM glucose but not in glucose-free media, indicating that increased glycolytic flux through glucokinase led to TNKS turnover.

### Non-glucose nutrients downregulate TNKS in INS-1 cells

The energetic effect of glucose on INS-1 cells is shared by other fuels such as pyruvate and amino acids [41]. We therefore investigated whether the TNKS-destabilizing effect of glucose was reproducible by pyruvate or by alanyl-glutamine, a dipeptide that releases glutamine upon hydrolysis by cellular peptidases [42]. Fig 3F shows that both fuels decreased TNKS abundance, supporting the notion that the energetic effect of diverse nutrients can stimulate TNKS turnover.

In addition to raising the energy charge in INS-1 cells, nutrients trigger a chain of distal events including membrane depolarization and calcium influx that culminate in insulin exocytosis [20]. We found that TNKS abundance in the presence of 3 mM glucose was not decreased by 30 mM KCl or 300 μM tolbutamide (to depolarize the membrane [41]), the calcium ionophore calcimycin (5 μM), or 20 μM insulin (to recapitulate the autocrine effect of insulin exocytosis)(data not shown), suggesting that these ATP-distal events alone do not stimulate TNKS turnover.

### Cell-type specificity of the glucose effect on TNKS PARsylation and turnover

**Fig 4:**
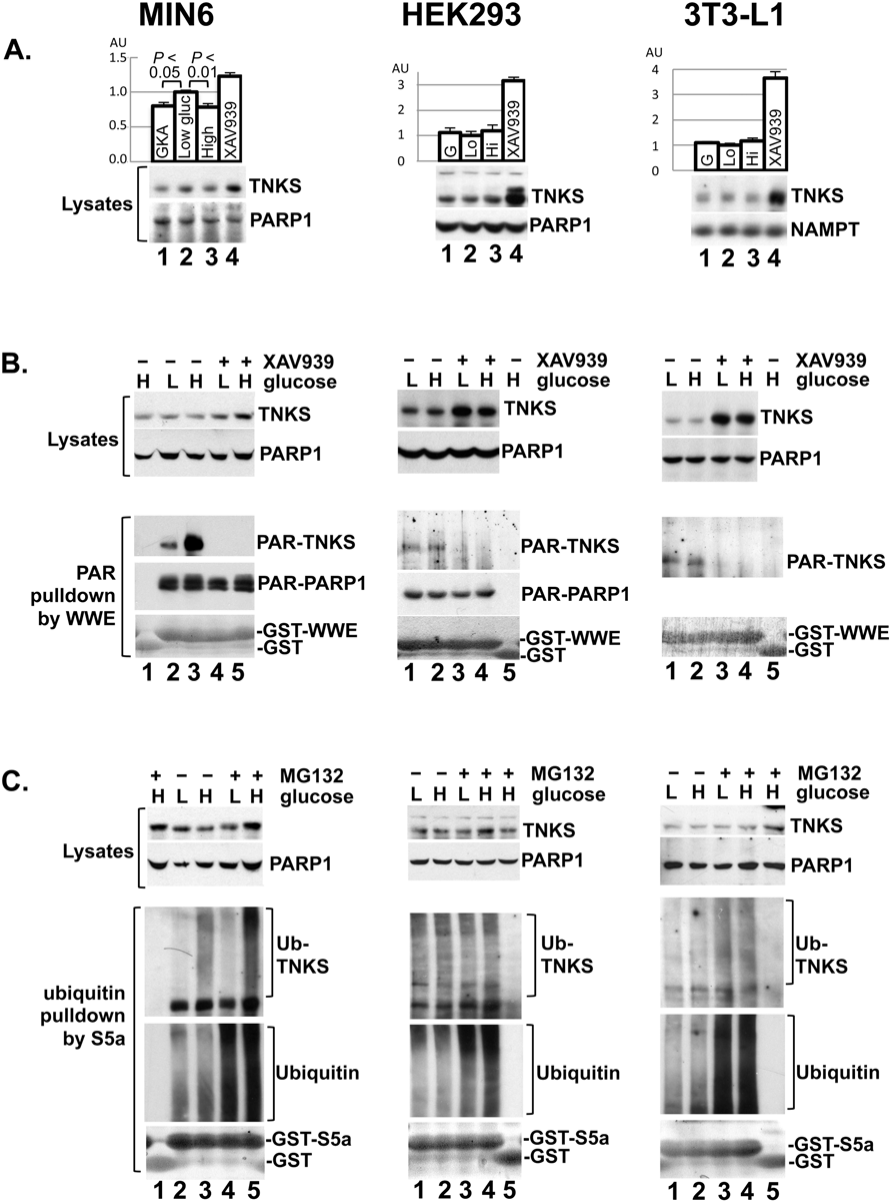
Cell-type specificity of the glucose effects on TNKS. **A.** Confluent cultures of mouse MIN6 insulinoma cells, human HEK293 renal epithelial cells, and mouse 3T3-L1 preadipocytes were treated in 24-well plates for 7 hr with glucokinase activator (10 μM GKA, lane 1) in 3 mM glucose, 3 mM glucose alone (lane 2), 14 mM glucose (lane 3), or XAV939 (4 μM, lane 4) in 3 mM glucose. Whole-cell extracts representing 10% of each well were immunoblotted for the indicated proteins. The immunoblots are representative of 2 independent experiments, each performed in 4 or 6 replicates per condition. The bar graphs indicate densitometer analysis of TNKS abundance (mean ± S.E.M). **B.** Confluent cultures of the indicated cell types were treated for 7 hr with 3 or 14 mM glucose as indicated by L (low) or H (high) in combination with XAV939 (4 μM) or DMSO. Lysates were incubated with GST as controls (lane 1 for MIN6; lane 5 for HEK293 and 3T3-L1) or with GST-WWE (15 μg, the remaining lanes) to precipitate PARsylated species as in Fig 3A. Lysates (upper panels) and the precipitates (lower panels) were immunoblotted for TNKS and PARP1. The precipitates were also Coomassie-stained for GST fusion proteins. Each lane represents a 15-cm plate (10 cm for MIN6 cells). PARsylated PARP1 was not detectable in 3T3-L1 cells. The data are representative of 2 or 3 experiments. **C.** Cells were treated with 3 (L) or 14 mM (H) glucose for 30 min before MG-132 (10 μM) or DMSO was added for another 30 min prior to harvesting for ubiquitination analysis as in Fig 3B. Each lane represents a 10-cm plate. The data are representative of 2 experiments, each performed in triplicate.

In contrast to insulin-secreting cells, most cells express low-*k*_m_ (high-affinity) hexokinases that are saturated at physiological levels of glucose, providing a stable glycolytic flux despite fluctuating ambient glucose levels. Therefore, the TNKS-destabilizing effect of glucose in INS-1 cells should be shared by other insulinoma lines like MIN6 cells but not unrelated cells such as HEK293 renal epithelial cells and 3T3-L1 preadipocytes. Indeed, high glucose levels and glucokinase activator both caused TNKS downregulation in MIN6 cells but not HEK293 or 3T3-L1 cells (Fig 4A). Moreover, only MIN6 cells exhibited a robust glucose-stimulated increase in TNKS PARsylation (Fig 4B) and ubiquitination (Fig 4C). Despite lacking a substantial glucose response, TNKS abundance in HEK293 and 3T3-L1 cells increased significantly following XAV939 treatment (Fig 4A). Collectively these data suggest that TNKS undergoes PAR-mediated degradation in diverse cell types, but this process is glucose-responsive only in insulin-secreting cells.

## Discussion

Using INS-1 as a ß cell model, this study shows for the first time that glucose and other metabolic fuels stimulate the NAD^+^-dependent catalytic activity of TNKS, thus establishing TNKS as a physiological sensor of cellular energy charge. By enhancing TNKS autoPARsylation and protein turnover, metabolic fuels bring about a decrease in TNKS abundance in ß cells. Our data suggest that nutritional regulation of TNKS activity and abundance is primarily mediated by cellular NAD^+^ content, which we have shown to expand in ß cells in response to glucose. The glucose effect on TNKS is conserved by MIN6, another ß cell line, but not HEK293 or 3T3-L1 cells.

Our NAD^+^ flux analyses show that INS-1 cells produce NAD^+^ primarily through NAMPT-mediated salvage of nicotinamide and that they turn over NAD^+^ with a half-life of about 5 hr (Fig 1A). Overall NAD^+^ consumption is reduced by approx. 20% by the TNKS inhibitor XAV939 (Fig 1B), suggesting that TNKS consumes a minor fraction of NAD^+^ in INS-1 cells. Interestingly, NAD^+^ production is accelerated by glucose treatment, leading to an expansion of NAD^+^ stores (Fig 2B). This glucose effect requires both NAMPT activity and mitochondrial function (Fig 2B) and is concomitant with glucose-induced increases in ATP levels (Fig 2A). Together with evidence that NAMPT is rate-limiting for NAD^+^ production *in vivo* [1,14] and is stimulated by ATP *in vitro* [16–19], our data indicate that glucose stimulates NAD^+^ production at least in part by raising cellular ATP levels to stimulate NAMPT.

Although glucose expands cellular NAD^+^ stores only modestly (Fig 2B), it robustly stimulates NAD^+^-dependent autoPARsylation of TNKS within hours (Figs 3A and S1). This disproportionality could be because overall NAD^+^ levels do not necessarily reflect the free (bioavailable) pool of NAD^+^ or the compartmentalized nature of its biosynthesis. Supporting this latter notion is that both TNKS [10] and the NAD^+^ biosynthesis enzyme NMNAT [43] are enriched on the cytosolic side of the Golgi membranes, where nascent NAD^+^ might be immediately funneled to TNKS without raising total NAD^+^ levels. It is also possible that NAD^+^-driven TNKS autoPARsylation is amplified by saturation of PAR glycohydrolase (PARG), which normally removes PAR from cellular proteins [44]. Alternatively, additional signals besides NAD^+^ may link cellular energy charge to TNKS catalytic activity. In any event, glucose stimulates robust autoPARsylation of TNKS, leading to PAR-dependent ubiquitination and degradation of TNKS (Fig 3). This TNKS-destabilizing effect of glucose is shared by other metabolic fuels (Fig 3F) and is reproducible by stimulating glucose utilization using a glucokinase activator (Fig 3E). The ability of TNKS catalysis to robustly reflect changes in cellular NAD^+^ metabolism is consistent with its *k*_m_ being above physiological NAD^+^ levels ([3,13] *vs*. [11,12]). Conversely, because cellular NAD^+^ levels are 10–20 times higher than the *k*_m_ of PARP1 [11], it is not surprising that glucose-induced increase in NAD^+^ does not translate into a substantial increase in PARP1 autoPARsylation (Figs 4B and S1).

The glucose effect on TNKS catalysis observed in INS-1 cells is shared by MIN6 cells (Fig 4B). Unlike INS-1 cells, MIN6 cells downregulate TNKS abundance only modestly in response to glucose treatment or glucokinase activation (Fig 4A *vs*. Fig 3). As potential explanations for this difference, it could be that TNKS exhibits a longer half-life in MIN6 cells and therefore requires prolonged treatment to manifest a substantial turnover. It could also be that a smaller fraction of TNKS undergoes glucose-stimulated PARsylation and turnover in MIN6 than in INS-1 cells. Regardless, both ß cell models share the ability to effectively couple glucose availability to TNKS catalysis.

Our finding that glucose and other nutrients expand NAD^+^ stores in ß cells (Fig 2B and 2C) goes against the paradigm that caloric restriction (CR) increases NAD^+^ levels [1,15]. In contrast to chronic CR models showing increased NAMPT abundance in muscle [1,15], our acute nutritional interventions in INS-1 cells had no discernable effects on NAMPT abundance (Fig 3). One possibility is that the CR-NAD^+^ paradigm established in muscle and fat does not apply all tissues [45]. In fact, the pancreas contains more NAD^+^ in the fed state than fasting [23]. Similarly, the NAD^+^-driven enzymes SIRT1 and SIRT4 in pancreatic ß cells are both more active in the fed state compared to starvation or CR [22,23]. Since feeding does not alter SIRT1 or SIRT4 abundance [22,23], their activation by feeding is likely attributable to greater NAD^+^ supply in the ß cells. The notion that feeding expands NAD^+^ stores appears applicable to the liver too, where a higher NAD^+^ content is found in the fed state than fasting [46,47]. Moreover, liver NAD^+^ content exhibits a nocturnal surge in *ad-lib* fed mice [12] but not fasted mice [48], again consistent with nutritional expansion of NAD^+^ stores. As a potential factor for why liver and ß cells deviate from the CR-NAD^+^ paradigm, both organs express glucokinase, the only hexokinase that is not saturated at physiological levels of glucose [40]. Consequently, as feeding raises plasma glucose levels, both organs are expected to increase glucose utilization and ATP production, culminating in ATP-mediated stimulation of NAMPT to synthesize NAD^+^.

Given that NAD^+^ is the obligatory co-substrate of TNKS, the diverse cellular processes regulated by TNKS catalysis [3–6] may be subject to nutritional regulation through the ATP → NAMPT → NAD^+^ pathway demonstrated here in β cells. Additional studies are required to further investigate the tissue specificity of this pathway, its potential superimposition on changes in NAMPT abundance induced by long-term nutritional intervention, and its impact on TNKS-regulated cellular processes like *wnt*/β-catenin signaling, telomere homeostasis, and energy metabolism. Because autoPARsylation of TNKS triggers ubiquitination [8], the ensuing proteasomal turnover may gradually offset the acute stimulation of TNKS catalysis by nutrients. We therefore speculate that acute surges in nutrients may be more efficient than chronic nutritional excess at stimulating TNKS catalysis.

## Supporting Information

S1 Fig Glucose treatment for 1 hr increases TNKS PARsylation in INS-1 cells. INS-1 cells were treated with 3 mM (lane 2) or 17 mM glucose (lanes 1 & 3) as in Fig 3A except that the treatment duration was 1 hour. Lysates were incubated as described in Materials and Methods with wild-type GST-WWE (15 mg, lanes 2–3) to precipitate PARsylated species, or with mutant GST-WWE (15 mg, lane 1) where an R163A substitution abolishes PAR binding [9] to serve as a negative control. The precipitates were immunoblotted for TNKS, PARP1, and WWE. All panels were from one experiment, which was representative of 3 independent studies.
(TIF)
