# Supplementary figures and images for: Nutritional Energy Stimulates NAD+ Production to Promote Tankyrase-Mediated PARsylation in Insulinoma Cells
Source: PLoS One. 2015 Apr 13;10(4):e0122948. doi: 10.1371/journal.pone.0122948 (PMC4395342; doi:10.1371/journal.pone.0122948)

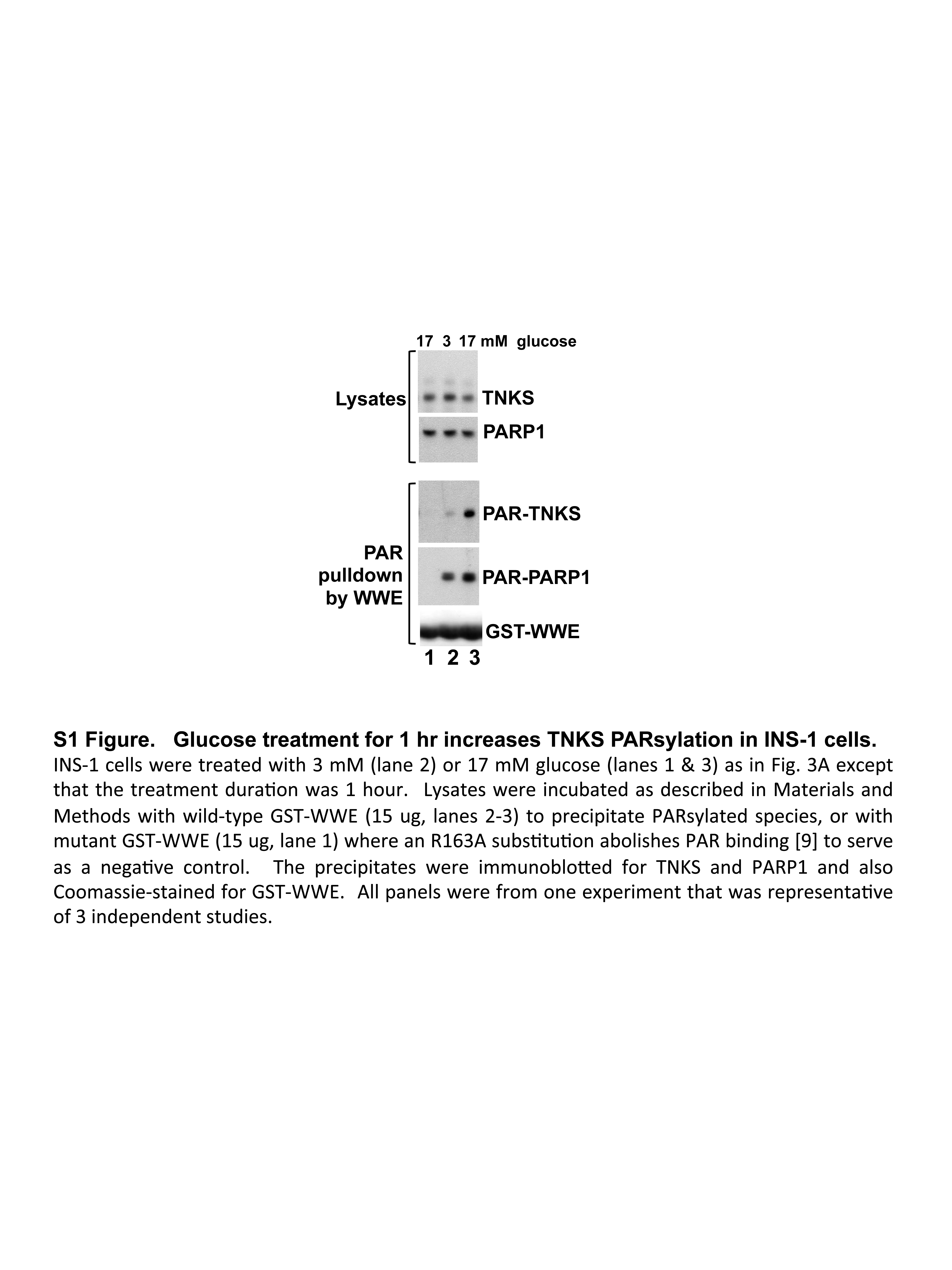

Supplement: S1 Fig — INS-1 cells were treated with 3 mM (lane 2) or 17 mM glucose (lanes 1 & 3) as in Fig 3A except that the treatment duration was 1 hour. Lysates were incubated as described in Materials and Methods with wild-type GST-WWE (15 mg, lanes 2–3) to precipitate PARsylated species, or with mutant GST-WWE (15 mg, lane 1) where an R163A substitution abolishes PAR binding [9] to serve as a negative control. The precipitates were immunoblotted for TNKS, PARP1, and WWE. All panels were from one experiment, which was representative of 3 independent studies. (TIF) [file pone.0122948.s001.tif]
